# ﻿A new species and a new provincial record of the genus *Acidota* Stephens from China (Coleoptera, Staphylinidae, Omaliinae)

**DOI:** 10.3897/zookeys.1173.102396

**Published:** 2023-08-07

**Authors:** Xi Chen, Yong-Qiang Xu, Zhong Peng

**Affiliations:** 1 Department of Biology, Shanghai Normal University, Shanghai, 200234, China Shanghai Normal University Shanghai China; 2 Tibet Plateau Institute of Biology, Lhasa, 540000, China Tibet Plateau Institute of Biology Lhasa China

**Keywords:** new provincial record, new species, rove beetles, taxonomic key

## Abstract

New taxonomic and faunistic data for two species of the genus *Acidota* Stephens, 1829 from China are provided. A new species from Xizang (Linzhi) is described and illustrated: *A.dawai* Peng & Chen, **sp. nov.** Additional data (including photographs of the habitus and the type labels) on the type specimens of taxa described from Japan (*A.crenatajaponica* Watanabe, 1990) and Taiwan (*A.montana* Smetana, 1993 and *A.nivicola* Smetana, 1993) are given. A key to Chinese species of *Acidota* is given. *Acidotacrenata* (Fabricius, 1792) is recorded from Heilongjiang for the first time.

## ﻿Introduction

Until now, eight species of the genus *Acidota* Stephens, 1829 have been reported from the Nearctic and Palaearctic regions (Smetana 1993; Assing 2002; Shavrin 2021). According to Shavrin (2019, 2021), the speciose omaliine genus *Acidota* is represented by seven described species in the Palaearctic region, three of which have been reported from China. Two species were described from Taiwan: *A.montana* Smetana, 1993 from Nenkaoshan and *A.nivicola* Smetana, 1993 from Hsuehshan (Smetana 1993). Shavrin (2019) recorded *A.crenata* (Fabricius, 1792) from Gansu, China.

This paper presents taxonomic and faunistic data for four Chinese species, including one new species (*Acidotadawai* Peng & Chen, sp. nov.) from Xizang and a new faunistic record of *A.crenata*. A key to the Chinese species of *Acidota* is provided. Additional data from the type of *A.crenatajaponica* Watanabe, 1990 is given.

## ﻿Material and methods

The examined material is deposited in the following public collections:

**SNUC** Insect Collection of Shanghai Normal University, Shanghai, China;

**ASC**Aleš Smetana Collection, the National Museum of Nature and Science, Toshiba, Japan.

The genitalia and other dissected parts were mounted on plastic slides and attached to the same pin as the respective specimens. Photographs were taken with a Canon EOS 7D camera with an MP-E 65 mm macro lens or with a Canon G9 camera mounted on an Olympus CX31 microscope.

The following abbreviations are used in the text, with all measurements in millimeters:

Total length (**TL**) length of body from anterior margin of mandibles (in resting position) to abdominal apex.

Length of forebody (**FL**) length of forebody from anterior margin of mandibles to posterior margin of elytra.

Head length (**HL**) length of head from anterior margin of frons to posterior constriction of head.

Head width (**HW**) maximum width of head.

Antenna length (AnL) length of antenna from the base to the apex.

Pronotum length (**PL**) length of pronotum along midline.

Pronotum width (PW) maximum width of pronotum.

Elytral length (EL) length at suture from apex of scutellum to elytral hind margin.

Elytral width (EW) combined width of elytra.

Length of aedeagus (**AL**) length of aedeagus from apex of ventral process to base of aedeagal capsule.

The type labels are cited in the original spelling; different labels are separated by slashes.

## ﻿Results

### ﻿ Acidota crenata

Taxon classificationAnimaliaColeopteraStaphylinidae

(Fabricius, 1792)

5A790DEA-E6EC-56DA-B29B-425DAB7EE5F8

Figs 1 , 2A

Staphylinus crenatus Fabricius, 1792: 525.
Acidota crenata : Stephens 1829: 25.
Omalium rufum Gravenhorst, 1802: 115.
Omalium castaneum Gravenhorst, 1806: 207.
Acidota pulchra Motschulsky, 1858: 493.
Acidota seriata LeConte, 1863: 55.
Acidota crenata japonica Watanabe, 1990: 145.

#### Type material.

***Paratype***: 1 ♂, labelled ‘Mt. Kurodake in Mts. Taisetsu, Hokkaido, Japan, 5–10.IX.1987, Coll. N. Yasuda / [PARATYPES] *Acidotacrenatajaponica* Y. Watanabe, 1990.’ (ASC).

#### Material studied.

China: 5 ♂♂, 1 ♀, Heilongjiang Prov., Huma County, Hongwei Town, alt. 580 m, 15.VII.2009, Li & Liu leg. (SNUC).

#### Additional material studied.

Canada: 1 ♂, ONT. 36 mi. S Pickle Lake, 22.VI.1973, Campbell & Parry (ASC).

#### Comment.

**Figure 1.**
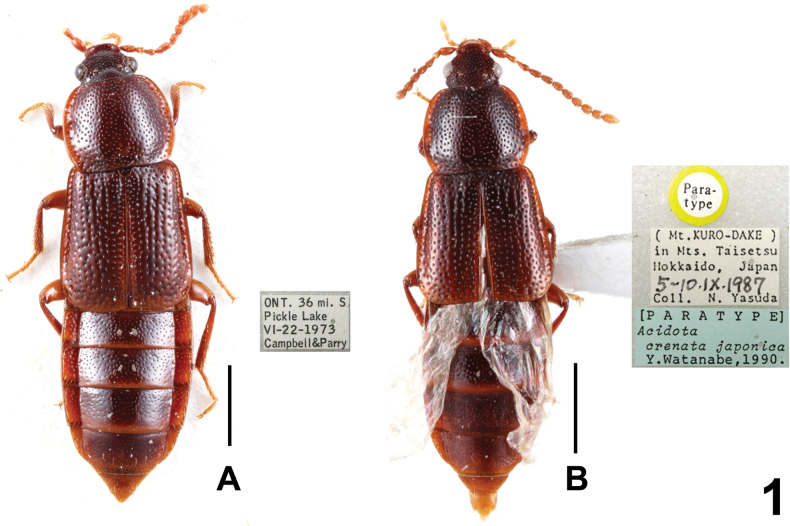
Habitus: **A***Acidotacrenata* (from Canada) **B***Acidotacrenatajaponica* (Paratype). Scale bars: 1.0 mm.

**Figure 2.**
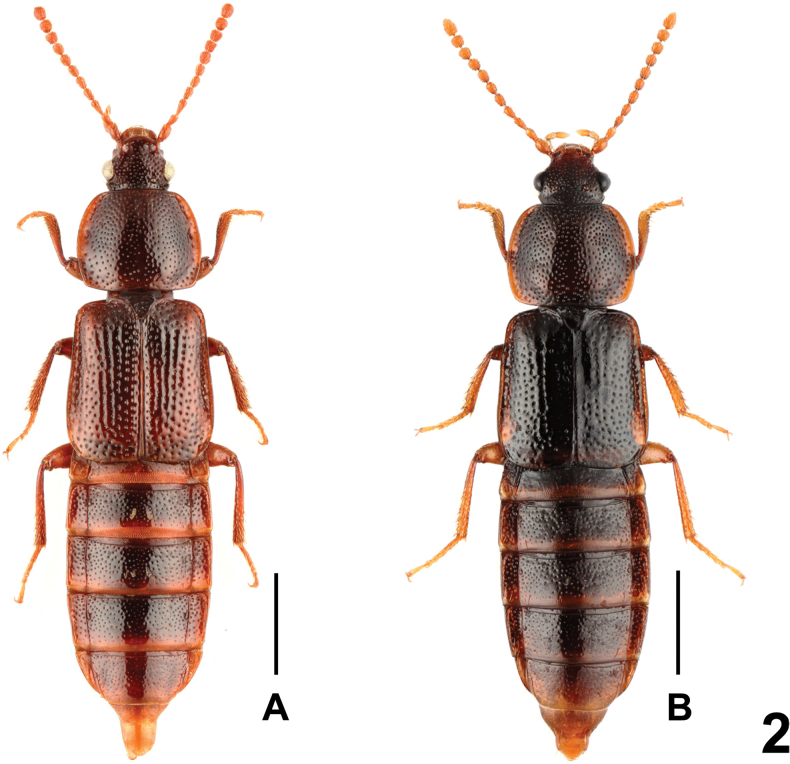
Habitus: **A***Acidotacrenata* (from China) **B***Acidotadawai*. Scale bars: 1.0 mm.

Habitus as in Figs 1, 2A. *Acidotacrenata* is one of the most widespread species of the genus. For illustrations of *A.crenata* (Fabricius, 1792) see Campbell (1982: figs 2, 4, 7, 10, 16, 19, 22, 25, 28, 31, 33, 34, 37, 40, 43), Smetana (1993: figs 4, 5) and Shavrin (2021: figs 1, 4–12). The above specimens (5 males and 1 female) from Heilongjiang represent new provincial records.

### ﻿ Acidota dawai

Taxon classificationAnimaliaColeopteraStaphylinidae

Z. Peng & X. Chen sp. nov.

11E1E129-3A8E-5AC5-BE82-A31F0CE8F763

https://zoobank.org/AC3EB7AE-4504-4E50-8582-864A52373D65

Figs 2B , 3

#### Type material.

***Holotype***: ♂, labelled ‘China: Xizang Prov., Linzhi City, Mt. Sejila, near Shejiema, 29°36’50”N 94°41’34”E, alt. 4340 m, 05.VII.2018, Chen, Peng & Shen leg.’ <white rectangular label, printed> / ‘HOLOTYPE: *Acidotadawai* sp. n., Peng & Chen des. 2023’ <red rectangular label, printed> (SNUC). ***Paratypes***: 5 ♂♂, 7 ♀♀: same data as holotype / ‘PARATYPE: *Acidotadawai* sp. n., Peng & Chen des. 2023’ <yellow rectangular label, printed> (SNUC).

#### Description.

Measurements (in mm) and ratios: TL: 6.00–6.91; FL: 3.15–3.60; HL: 0.43–0.46; HW: 0.70–0.74; AnL: 1.56–1.75; PL: 0.93–0.98; PW: 1.20–1.24; EL: 1.22–1.33; EW: 1.32–1.42; AL: 0.77–0.84; HW/HL: 1.59–1.64; HW/PW: 0.58–0.60; HL/PL: 0.46–0.48; PW/PL: 1.26–1.28; EL/PL: 1.27–1.34.

***Body*** (Fig. 2B) blackish brown, antennae paler, basal portions of femora, apical portions of tibiae and tarsi brown to light brown.

***Head*** subtriangular, distinctly transverse; clypeus convex; eyes very convex, about 1.73 times as long as temples (holotype); ocelli distinct, distance between ocelli 1.9 times as long as distance between ocellus and posterior margin of eye (holotype). Punctation of forebody coarse and dense; pubescence moderately long and dense. Antennae (Fig. 1C) slender, length × width (in mm) of antennomeres 1–11 (holotype): 0.20 × 0.10: 0.14 × 0.07: 0.14 × 0.07: 0.14 × 0.07: 0.13 × 0.08: 0.13 × 0.08: 0.13 × 0.08: 0.13 × 0.10: 0.11 × 0.11: 0.11 × 0.12: 0.18 × 0.12.

***Pronotum*** slightly transverse, widest in the middle; disc convex, without impression; punctures similar to that of head, but more distinct; pubescence moderately long and dense.

***Elytra*** slightly convex, 1.1 times as wide as long; punctation coarser and sparser than that of pronotum; pubescence distinctly sparser than that of pronotum. Hind wings well developed.

***Abdomen*** slender, widest at segment V, evenly narrowing posteriorly. Abdominal tergites with fine and dense punctation, and short decumbent pubescence, denser on apical tergites; tergites IV–V with a pair of tomentose spots in middle, spots on tergite V smaller and less transverse.

**Figure 3.**
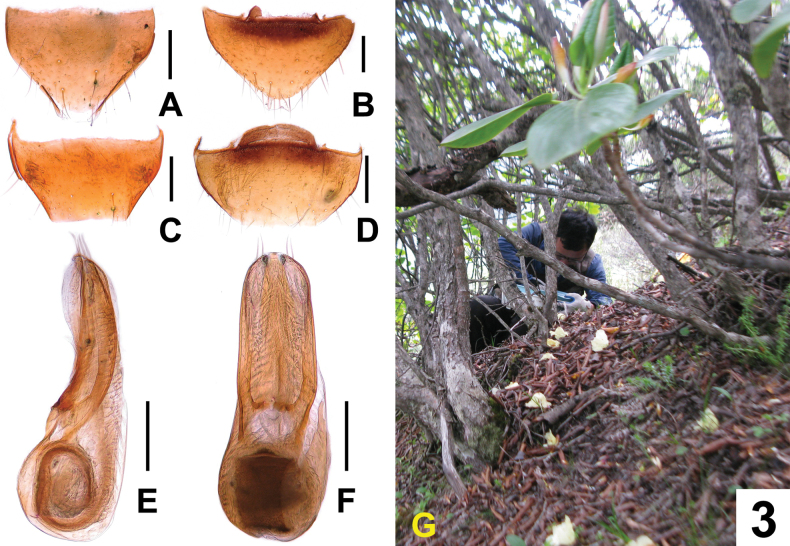
*Acidotadawai***A** female tergite VIII **B** female sternite VIII **C** male tergite VIII **D** male sternite VIII **E** aedeagus in lateral view **F** aedeagus in ventral view **G** Zhi-Fei Cheng collecting *Acidotadawai* at Mt. Sejila, Xizang. Scale bars: 0.2 mm.

**Male.** Posterior margin of abdominal tergite VIII (Fig. 3C) and sternite VIII (Fig. 3D) truncate. Aedeagus as in Fig. 3E, F; median lobe indistinctly narrowed toward moderately wide with subacute apex; parameres symmetrical, wide, reaching apex of median lobe, each bearing two apical setae; internal sac wide and long, spirally folded in basal portion.

**Female.** Posterior margin of abdominal tergite VIII (Fig. 3A) somewhat truncate. Posterior margin of abdominal sternite VIII (Fig. 3B) rounded.

#### Distribution and natural history.

The type locality is situated in the Sejila Mountain to the east of Linzhi, south-eastern Xizang. Some of the specimens were sifted from rhododendron litter and humus in a rhododendron forest on a west slope near the mountain summit at an altitude of 4340 m (Fig. 3G).

#### Etymology.

This species is dedicated to Mr Dawa, who supported us on our field trips.

#### Comparative notes.

Regarding the general shape of the body, aedeagus, and features of the punctation and pubescence, *A.dawai* is similar to dark-coloured specimens of the morphologically variable *A.crenata*, a widespread species in the Holarctic region. The new species can be distinguished from *A.crenata* by the distinctly more transverse head, longer antennomere 2, and the shapes of the slightly wider and shorter parameres, which are subparallel in the middle and gradually narrowing towards the apex (parameres of *A.crenata* are insignificantly narrower, moderately strongly narrowing apicad).

### ﻿ Acidota montana

Taxon classificationAnimaliaColeopteraStaphylinidae

Smetana, 1993

68362B44-8B16-5F97-994C-2D3392F87E49

Fig. 4A

Acidota montana Smetana, 1993: 71.

#### Type material.

***Holotype***: ♂, labelled ‘TAIWAN, Nantou Hsien, Nenkaoshan 2.5 km SW Tenchi Hut, 2720 m, 6.V.92 A. Smetana [T115] / [HOLOTYPE] *Acidotamontana* A. Smetana, 1992.’ (ASC).

#### Comment.

**Figure 4.**
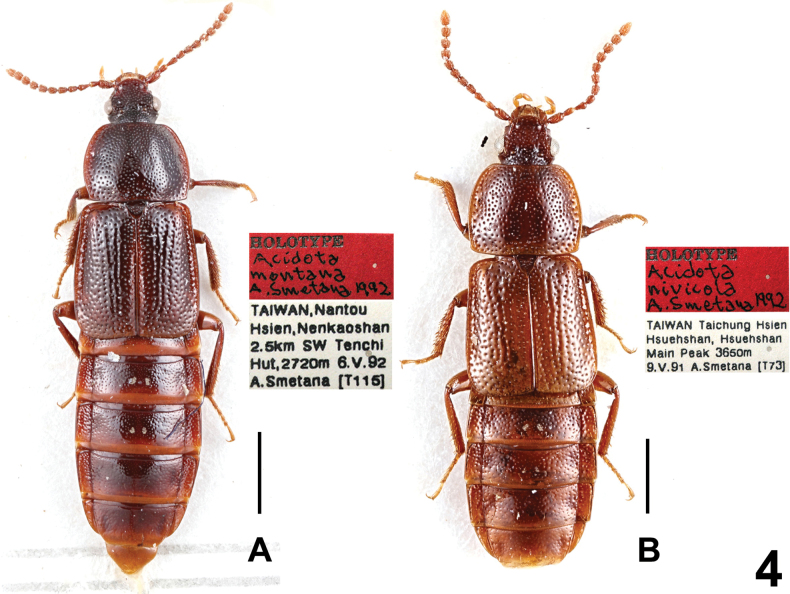
Habitus: **A***Acidotamontana* (Holotype) **B***Acidotanivicola* (Holotype). Scale bars: 1.0 mm.

Habitus as in Fig. 4A. This species is known only from Taiwan (Smetana, 1993). For illustrations of *A.montana* see Smetana (1993: figs 1–3).

### ﻿ Acidota nivicola

Taxon classificationAnimaliaColeopteraStaphylinidae

Smetana, 1993

942A0442-6A74-504B-881C-433015155EA3

Fig. 4B

Acidota nivicola Smetana, 1993: 74.

#### Type material.

***Holotype***: ♂, labelled ‘TAIWAN Taichung Hsien Hsuehshan, Hsuehshan Main Peak 3650 m, 9.V.91 A. Smetana [T 73] / [HOLOTYPE] *Acidotanivicola* A. Smetana, 1992.’ (ASC).

#### Comment.

Habitus as in Fig. 4B. This species is known only from Taiwan (Smetana, 1993). For illustrations of *A.nivicola* see Smetana (1993: figs 6, 7).

### ﻿Key to the *Acidota* species of China

**Table:** 

1	Smaller species (length of body: 4.5 mm). Aedeagus narrower, apex of median lobe narrowly subtruncate. Habitus as in Fig. 4B. Taiwan	***A.nivicola* Smetana, 1993**
–	Larger species (length of body ≥ 5.0 mm). Aedeagus more robust, apex of median lobe subacute	**2**
2	Pronotum distinctly wider than long (PW/PL ≥ 1.35), more sharply narrowing posteriad, with obtusely angulate hind angles. Habitus as in Fig. 4A. Taiwan	***A.montana* Smetana, 1993**
–	Pronotum slightly transverse (PW/PL < 1.30), gradually narrowing posteriad, with obtuse to rounded hind angles	**3**
3	Head slightly transverse. Antennomere 2 of the antennae distinctly shorter than antennomere 3. Parameres slightly longer, exceeding apex of median lobe, from middle moderately strongly narrowing apicad. Habitus as in Figs 1A, 1B, 2A. Holarctic region	***A.crenata* (Fabricius, 1792)**
–	Head distinctly transverse. Antennomere 2 of the antennae nearly as long as antennomere 3. Parameres slightly shorter, reaching apex of median lobe, subparallel in middle, from apical portion gradually narrowing apicad (Fig. 3F). Habitus as in Fig. 2B. China: Xizang	***A.dawai* sp. nov.**

## Supplementary Material

XML Treatment for Acidota crenata

XML Treatment for Acidota dawai

XML Treatment for Acidota montana

XML Treatment for Acidota nivicola

## ﻿Additional information

### Data availability

All of the data that support the findings of this study are available in the main text or Suppl. material 1.

## Supplementary materials

Supplementary material 1
Acidotadawai
Data type COL Explanation note Body blackish brown, antennae paler, basal portions of femora, apical portions of tibiae and tarsi brown to light brown. Head subtriangular and transverse, broadest at level of eyes; clypeus convex; eyes prominent, about 1.73 times as long as temples (holotype); ocelli distinct, distance between ocelli 1.9 times as long as distance between ocellus and posterior margin of eye (holotype). punctures coarse and dense; pubescence moderately long and dense. Antennae slender, length × width (in mm) of antennomeres 1–11 (holotype): 0.20 × 0.10 : 0.14 × 0.07 : 0.14 × 0.07: 0.14 × 0.07 : 0.13 × 0.08 : 0.13 × 0.08 : 0.13 × 0.08 : 0.13 × 0.10 : 0.11 × 0.11 : 0.11 × 0.12 : 0.18 × 0.12. Pronotum slightly transverse, widest in the middle; disc convex, without impression; punctures similar to that of head, but more distinct; pubescence moderately long and dense. Elytra slightly convex, 1.1 times as wide as long; punctures coarser and sparser than those of pronotum; pubescence distinctly sparser than those of pronotum. Hind wings present. Abdomen slender, widest at segment V, evenly narrowing posteriorly. Tergites with fine and dense punctures, and short decumbent pubescence, denser on apical tergites; tergites IV–V with a pair of tomentose spots in middle, spots on tergite V smaller and less transverse. Male. Posterior margin of tergite VIII and sternite VIII truncate; median lobe of aedeagus indistinctly narrowed toward moderately wide with subacute apex; parameres symmetrical, significantly exceeding apex of median lobe, each bearing two apical setae; internal sac with one moderately narrow and very long flagellum. Female. Posterior margin of tergite VIII and sternite VIII strongly convex. https://binary.pensoft.net/file/889176This dataset is made available under the Open Database License (http://opendatacommons.org/licenses/odbl/1.0/). The Open Database License (ODbL) is a license agreement intended to allow users to freely share, modify, and use this Dataset while maintaining this same freedom for others, provided that the original source and author(s) are credited.Xi Chen, Zhong Peng
