# Supplementary figures and images for: ﻿A new species and a new provincial record of the genus Acidota Stephens from China (Coleoptera, Staphylinidae, Omaliinae)
Source: Zookeys. 2023 Aug 7;1173:297–305. doi: 10.3897/zookeys.1173.102396 (PMC10425872; doi:10.3897/zookeys.1173.102396)

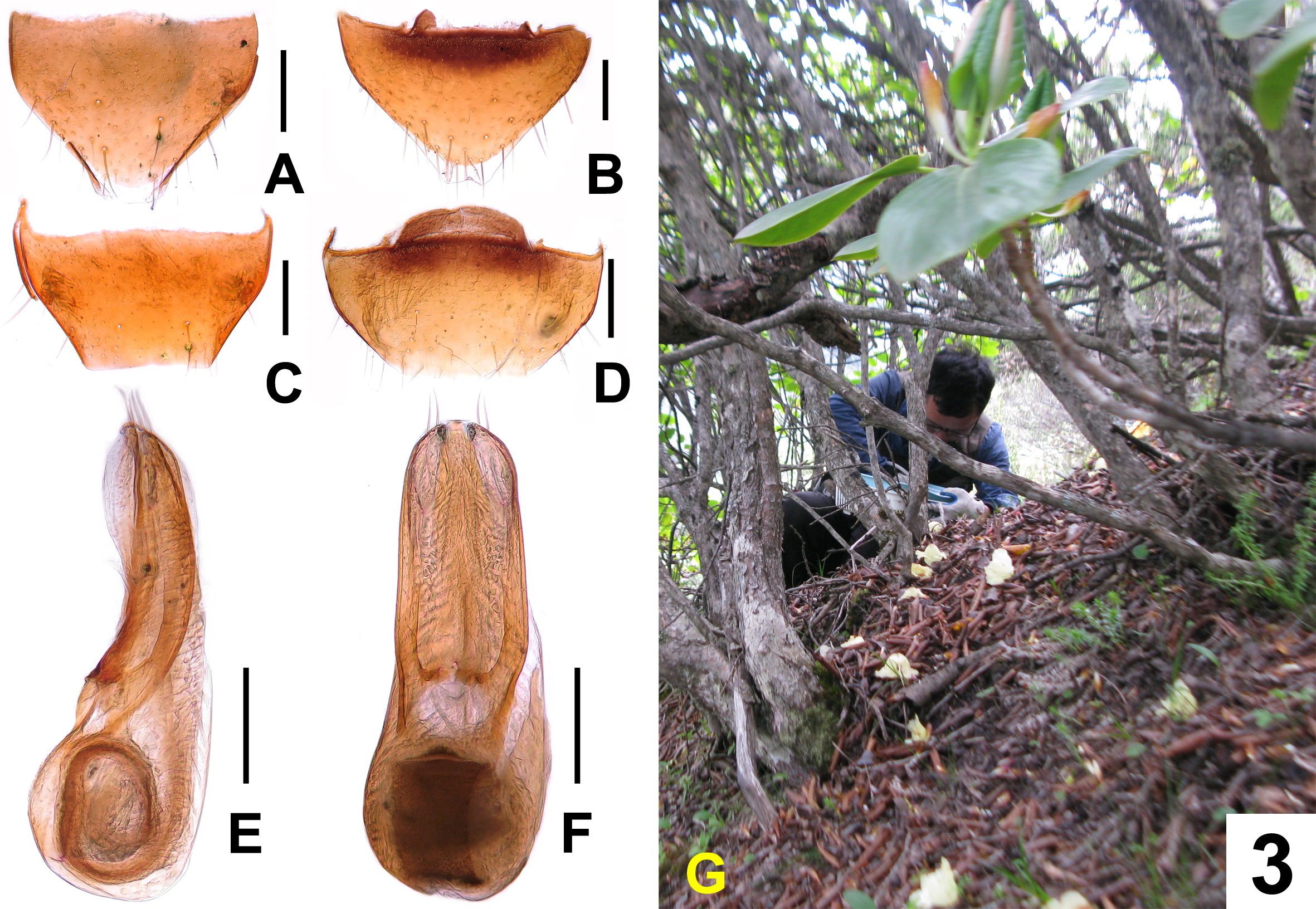

Supplement: Supplementary material 1 — Acidotadawai [file zookeys-1173-297_article-102396__-s001.jpg]
